# Genomic Instability Is an Early Event in Aluminium-Induced Tumorigenesis

**DOI:** 10.3390/ijms21239332

**Published:** 2020-12-07

**Authors:** Stefano J. Mandriota, Mirna Tenan, Adeline Nicolle, Julia D. Jankowska, Paolo Ferrari, Jean-Christophe Tille, Mary-Anne Durin, Catherine M. Green, Sebastien Tabruyn, Daniela Moralli, André-Pascal Sappino

**Affiliations:** 1 Laboratoire de Cancérogenèse Environnementale, Fondation des Grangettes, 1224 Chêne-Bougeries, Switzerland; mirna@alucancerlab.com (M.T.); adeline@alucancerlab.com (A.N.); paolo.ferrari@fht.org (P.F.); pascal.sappino@grangettes.ch (A.-P.S.); 2 Wellcome Centre for Human Genetics, University of Oxford, Oxford OX3 7BN, UK; 1714759@brunel.ac.uk (J.D.J.); mary-anne.durin@rdm.ox.ac.uk (M.-A.D.); catherine.green@well.ox.ac.uk (C.M.G.); dmoralli@well.ox.ac.uk (D.M.); 3 Department of Pathology, Geneva University Hospital, 1205 Geneva, Switzerland; jean-christophe.tille@hcuge.ch; 4 TransCure bioServices, 74160 Archamps, France; sebastien.tabruyn@tcbioservices.com

**Keywords:** aluminium, breast cancer, carcinogenesis, genomic instability, clastogens

## Abstract

Genomic instability is generally considered as a hallmark of tumorigenesis and a prerequisite condition for malignant transformation. Aluminium salts are suspected environmental carcinogens that transform mammary epithelial cells in vitro through unknown mechanisms. We report here that long-term culture in the presence of aluminium chloride (AlCl_3_) enables HC11 normal mouse mammary epithelial cells to form tumours and metastases when injected into the syngeneic and immunocompetent BALB/cByJ strain. We demonstrate that AlCl_3_ rapidly increases chromosomal structural abnormalities in mammary epithelial cells, while we failed to detect direct modulation of specific mRNA pathways. Our observations provide evidence that clastogenic activity—a well-recognized inducer of genomic instability—might account in part for the transforming abilities of aluminium in mammary epithelial cells.

## 1. Introduction

Breast cancer incidence is largely dependent on environmental factors that remain largely unidentified. It has been rising in Western societies starting from the second half of the 1960s. Salts of aluminium, a versatile chemical element devoid of physiological function, have long been suspected to contribute to this phenomenon. In addition to antiperspirants, where it is added because of its capacity to prevent sweating, aluminium is present in many other industrial products of frequent use, including sunscreens, food additives, anti-acid drugs and vaccines [1,2,3].

Epidemiological studies investigating the link between aluminium and breast cancer suggest an association between the incidence of breast cancer and the use of aluminium containing antiperspirants at a young age [4,5]. Such studies are limited by their retrospective nature, by small cohort size and by the lack of a proper control group, due to the general exposure to aluminium in our societies.

Concentrations of aluminium in the range of those measured in the breast of women living in industrialized countries (0.8–87 μM) (reviewed in [6]) transform MCF-10A human mammary epithelial cells and NMuMG mouse mammary epithelial cells in vitro [7,8]. When injected into NOD-SCID or nude mice, NMuMG cells transformed in vitro by aluminium form tumours and metastasis [8]. In other studies, aluminium increased the migratory and invasive properties of MCF-7 or MDA-MB-231 human breast cancer cells in vitro [9,10]. The transforming effect of aluminium appeared to be restricted to mammary epithelial cells [7]. In MCF-10A cells, the transforming effects of aluminium were preceded by the induction of DNA double strand breaks (DSB) [7]. The induction of DSB by aluminium was also observed in primary human mammary epithelial cells [7], in embryonic zebrafish cells [11] and in *Arabidopsis thaliana* [12]. In these studies, the induction of DSB by aluminium was assessed by phosphorylated histone H2AX (γ-H2AX) immunofluorescence.

Aluminium is not detectably mutagenic in the Ames test, which mainly detects single base mutations in *Salmonella typhimurium* [7]. This result might reflect intrinsic differences in mutagenesis in bacteria vs. eukaryotic cells. However, it also raises the possibility that DSB observed in aluminium-treated cells may result in mutations other than single base substitutions.

In this study, we extended our investigations on the transforming effects of aluminium [7,8] to HC11 cells, a spontaneously immortalized, non-tumorigenic mouse mammary epithelial cell line isolated from BALB/c mice [13]. In contrast to NMuMG cells, HC11 cells can be used in syngeneic graft experiments, since immunocompatible mouse strains are available. We build upon our past results from aluminium-induced transformation of NMuMG cells [8] and on new evidence that aluminium transforms HC11 mammary epithelial cells in vitro (this study) to show that, at concentrations in the range of those measured in the human mammary gland, aluminium induces genomic instability in mammary epithelial cells.

## 2. Results

### 2.1. AlCl_3_ Transforms HC11 Mouse Mammary Epithelial Cells In Vitro

**Figure 1:**
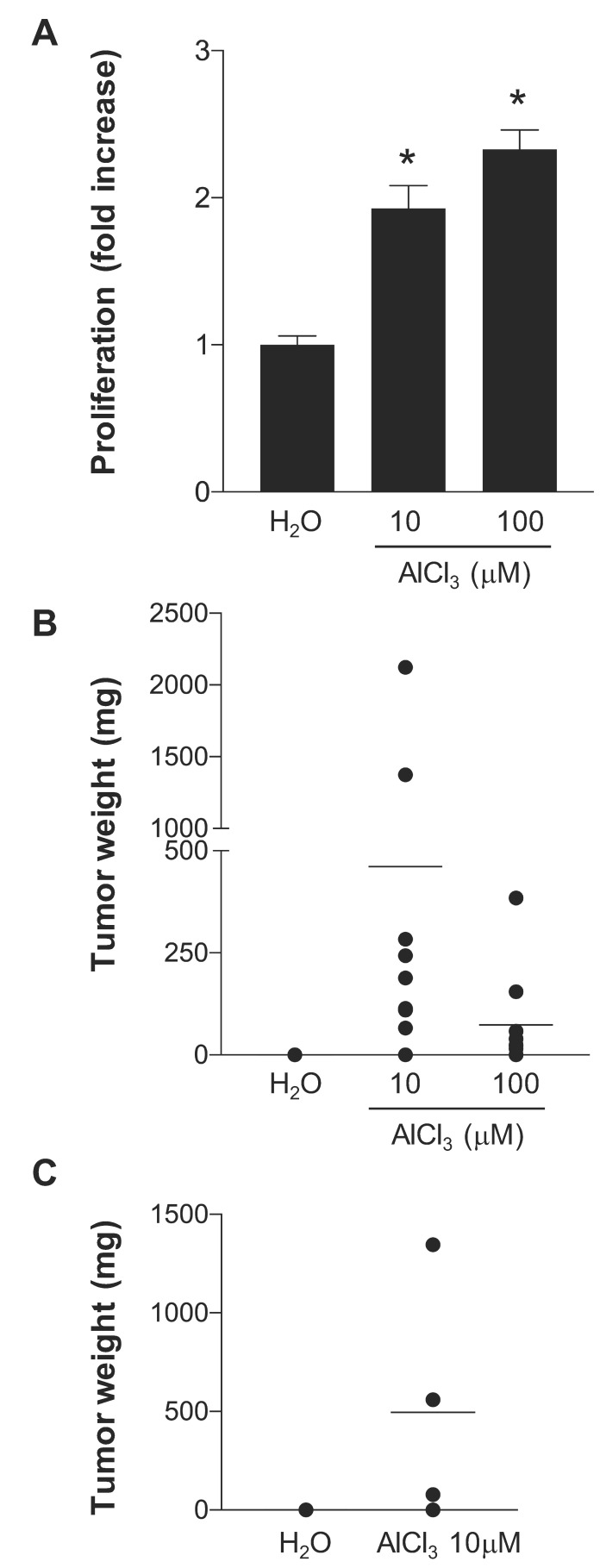
AlCl_3_ transforms HC11 cells in vitro. (**A**) HC11 cells cultured for 70–73 weeks in the presence of the indicated concentrations of AlCl_3_ or the same dilution (1/1000) of solvent (H_2_O) alone were seeded in triplicate in ultra-low attachment 6-well plates at the density of 3 × 10^4^ cells/well and grown in complete medium—in the absence of AlCl_3_—for 6 days. At the end of the incubation, cell proliferation was measured as reported in Materials and Methods. Data presented in the graph are means +/− SEM from three independent experiments. * *p*-value AlCl_3_ *vs.* H_2_O < 0.0001 (Dunnett’s multiple comparisons test) for both AlCl_3_ concentrations. (**B**) HC11 cells cultured for 71 weeks in the presence of the indicated concentrations of AlCl_3_, or the same volume (1/1000) of solvent (H_2_O) alone were injected subcutaneously into the flank of 6-week-old BALB/cByJ female mice. Mice were inspected twice a week for tumour formation and sacrificed five months after injection. The graph shows the weight of each individual tumour at the time of dissection. A total of ten mice/condition were used, in two independent experiments. (**C**) HC11 cells cultured for 71 weeks in the presence of AlCl_3_ 10 μM, or the same volume (1/1000) of solvent (H_2_O) alone were injected into the mammary fat pad of the right ventral nipple of 6 weeks old BALB/cByJ female mice. Mice were sacrificed five months after injection. The graph shows the weight of each individual tumour at the time of dissection. Five mice or four mice were used for the H_2_O or AlCl_3_ 10 μM conditions, respectively.

We previously reported that long-term culture in the presence of 10–300 μM AlCl_3_ transforms MCF10A human mammary epithelial cells or NMuMG mouse mammary epithelial cells [7,8]. The 10–300 μM concentration range overlaps with the aluminium content measured in the human mammary gland of women living in industrialized countries (0.8–87 μM) [6]. Chlorides of gallium or indium that, as aluminium, belong to the thirteenth group of the Mendeleev Table and hence have similar chemical properties had no effect [7]. NMuMG cells transformed in vitro by AlCl_3_, but not untreated controls cultured in parallel, formed tumours and metastasis in NOD-SCID and nude mice [8]. In those experiments, we used immunodeficient mice because the NAMRU strain, from which NMuMG cells were originally isolated, is no longer available. Therefore, those results do not say whether aluminium can enable mammary epithelial cells to form tumours in an immunocompetent animal system. This is an important point, since the majority of tumours develop in the presence of a functional immune system. To investigate this point, we assessed the capacity of aluminium to transform HC11 cells, a spontaneously immortalized mammary epithelial cell line originally isolated from BALB/c mice [13], for which immunocompatible mouse strains exist. After approximately seventy weeks of culture in the continuous presence of AlCl_3_ 10 μM or 100 μM, HC11 cells exhibit loss of contact inhibition compared to HC11 cells cultured in parallel in the presence of the same dilution (1/1000) of solvent (H_2_O) alone (Figure S1). This is consistent with the loss of contact inhibition and induction of epithelial–mesenchymal transition (EMT) in MCF-10A or NMuMG cells chronically cultured in the presence of the same concentrations of AlCl_3_ [7,8]. In the growth-in-low-attachment (GILA) assay that assesses cellular transformation in vitro by measuring anchorage-independent proliferation [14], HC11 cells cultured in the presence of AlCl_3_ exhibited a dose-dependent increase in proliferation compared to controls (Figure 1A).

In four- or seven-days proliferation experiments, AlCl_3_ did not increase proliferation of HC11 parental cells. Rather, at the concentration of 100 μM it slightly decreased HC11 cell proliferation after 7 days compared to controls (Figure S2A,B left panels). This is consistent with our previous observations in NMuMG and MCF-10A parental cells [7,8]. In four- or seven- days proliferation experiments, AlCl_3_ had little or no effect on HC11 cell survival (Figures S2A,B right panels and S2C).

Taken together, these experiments show that chronic culture in the presence of AlCl_3_ enhance anchorage-independent grow—an indicator of cellular transformation—in HC11 cells. They also suggest that this effect is not mediated by a direct mitogenic effect of aluminium, since in four- or seven-days incubation experiments, aluminium rather slightly decreases than increases HC11 cell proliferation. The latter effect occurred in the absence of detectable cytotoxicity, which might reflect slight inhibition of proliferation or some form of cellular stress.

HC11 cells transformed in vitro by chronic AlCl_3_ exposure or controls cultured in parallel (Figure 1A) were injected subcutaneously into the flank of BALB/cByJ mice. This immunocompetent mouse strain shares major histocompatibility genes with BALB/c mice—the strain the HC11 cells were originally isolated from—no longer available. In two independent experiments, a total of 9/10 mice injected with HC11 cells transformed in vitro by AlCl_3_ 10 μM, and a total of 8/10 mice injected with HC11 cells transformed in vitro by AlCl_3_ 100 μM, formed palpable tumours at the site of injection, whereas none of the ten mice injected with control HC11 cells cultured in parallel formed palpable tumours over the time period considered. The two largest tumours were observed in the group injected with HC11 cells transformed in vitro by AlCl_3_ 10 μM (Figure 1B and Figure S3A). However, the difference in tumour size between the AlCl_3_ 10 μM group and the AlCl_3_ 100 μM group was not statistically significant.

**Figure 2:**
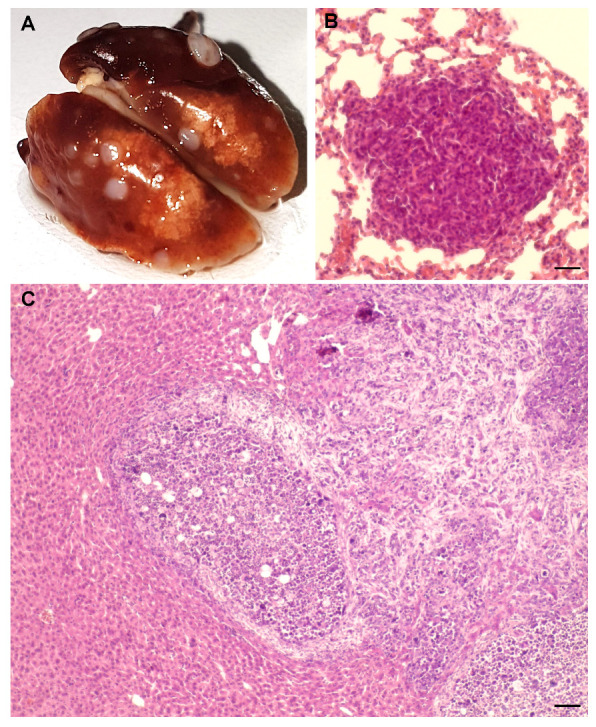
AlCl_3_ promotes HC11 cell tumour metastasis in syngeneic immunocompetent mice. (**A**) Whole lungs at the time of dissection or (**B**) hematoxylin/eosin (HE) staining of a lung metastasis found in a BALB/cByJ female mouse injected subcutaneously with HC11 cells transformed in vitro by AlCl_3_ 10 μM (see Figure 1B). (**C**) HE staining of a hepatic metastasis in a Balb/cByJ female mouse injected orthotopically with HC11 cells transformed in vitro by AlCl_3_ 10 μM (see Figure 1C). Bar = (**B**) 50 μm; (**C**) 100 μm.

By histology, the tumours were invasive carcinomas, deeply infiltrating the hypodermis at the site of injection and the underlying smooth muscle cell layer. Necrotic areas were present in the majority of the tumours. At the cellular level, tumours consisted of poorly differentiated cells characterized by a complete loss of epithelial polarity, mesenchymal morphology, a high nucleus/cytoplasm ratio with numerous mitosis, and nuclear heterogeneity. Tumours had a Ki67 proliferation index of approximately 40%. Figure S3B–D provide examples of these results. Metastasis were observed in the lungs of the two mice carrying the two largest tumours (Figure 2A,B). One of them also presented with splenomegaly at the time of dissection.

Histological analysis of the injection site confirmed the complete absence of tumour growth in 10/10 mice injected with control HC11 cells.

In a separate experiment, HC11 cells transformed in vitro by AlCl_3_ 10 μM, or their controls cultured in parallel, were injected into the mammary fat pad of the right ventral nipple of 6-week-old BALB/cByJ female mice. Three of four mice injected with AlCl_3_ 10 μM-transformed cells developed palpable tumours at the site of injection (Figure 1C). The mouse carrying the largest tumour presented with splenomegaly, tumour infiltration in the peritoneum and right leg, partial destruction of the right femur by the tumour and a large hepatic metastasis (Figure 2C). Histological features of the tumours were similar to those of the tumours grown subcutaneously (see above). None of the five mice injected with control HC11 cells developed palpable tumours over the time period considered (Figure 1C). Histological analysis of the site of injection in the latter mice confirmed the complete absence of tumour growth.

Taken together, our results with HC11 cells confirm and extend our previous results with MCF-10A and NMuMG cells, the compelling novelty presented in this study being that HC11 cells transformed in vitro by aluminium form tumours and metastasis in an immunocompetent animal model.

To explore the mechanisms that might underlie mammary epithelial cell transformation by AlCl_3_, we performed the following analyses.

### 2.2. Analysis of mRNA Expression Patterns in AlCl_3_-Treated HC11 or NMuMG Cells

The mRNA profiling of mammary epithelial cells transformed in vitro by AlCl_3_ might provide insights into the mechanisms by which this salt transforms this cell type. So far, it has not been established. To investigate this point, we analysed the gene expression profile of HC11 cells or NMuMG cells transformed in vitro by AlCl_3_, or the respective, untreated controls, by a cDNA microarray covering approximately 20,800 well-annotated genes. We used the following three independent sets of cultures: (a) HC11 cells transformed in vitro by AlCl_3_ 10 μM, 100 μM, or untransformed controls cultured in parallel, used for the syngeneic graft experiments reported above; (b) NMuMG cells transformed in vitro by AlCl_3_ 100 μM, or untransformed controls cultured in parallel, previously reported [8] and referred to as NMuMG series I in this study; (c) a new set of NMuMG cells transformed in vitro by AlCl_3_ 100 μM, or untransformed controls cultured in parallel, referred to as NMuMG series III in this study (Figure S4). We considered genes whose mRNA levels were at least four-fold higher, or four-fold lower, in HC11 or NMuMG cells transformed in vitro by AlCl_3_, than in the respective controls. Statistic cut-off was set at false discovery rate (FDR) *p* value < 0.05.

We started by considering the gene expression profile of HC11 or NMuMG cells transformed in vitro by AlCl_3_ 100 μM, since this condition is available for the three sets of cultures. In HC11 cells, we found 421 genes regulated by at least four-fold in AlCl_3_-transformed cells compared to controls. In NMuMG series I, the number of such genes was 398 and in NMuMG series III, 566. These cDNA microarrays were registered in ArrayExpress with accession numbers E-MTAB-9723, E-MTAB-9733 or E-MTAB-9735, respectively. We reasoned that being both mammary epithelial in nature, both of mouse origin, and both transformed in vitro by AlCl_3_, HC11 and NMuMG cells can be hypothesized to have similar transcriptional responses to aluminium, if these exist. When looking for genes regulated in cells transformed in vitro by AlCl_3_ 100 μM *vs.* the respective untransformed controls in the three series of cells, we found twenty-one common genes. Of these, however, only three were regulated in the same direction (positively or negatively) in the three series. These were an upregulated gene, *Sulf2*, and two downregulated genes, *Hsd11b1* and *Enpp5* (Excel File S1A,B). These three genes were also consistently deregulated in HC11 cells transformed in vitro by AlCl_3_ 10 μM (see ArrayExpress E-MTAB-9723). cDNA microarray data were validated by quantitative real-time PCR (Table S1A,B). Of these genes, *Sulf-2* is an extracellular sulfatase reported as a positive regulator of oncogenic signalling pathways (see Discussion), whereas *Hsd11b1* or *Enpp5* encode corticosteroid 11-beta-dehydrogenase isozyme 1 or ectonucleotide pyrophosphatase/phosphodiesterase family member 5, respectively, two enzymes with little or no reported evidence of involvement in cellular transformation.

The difference in *Sulf-2*, *Hsd11b1* or *Enpp5* mRNA levels observed in AlCl_3_-transformed HC11 or NMuMG cells vs. the respective controls might represent a direct regulatory effect of AlCl_3_, or occur indirectly, as a secondary effect of cellular transformation. To distinguish between these possibilities, we performed a second mRNA analysis where HC11 cells or NMuMG cells were incubated in the continuous presence of AlCl_3_ 100 μM, or the equivalent volume of H_2_O as a control, for 4 weeks, a duration that precedes cellular transformation [7,8]. Rather than limiting our investigations to the *Sulf2*, *Enpp5* and *Hsd11b1* genes, in this experiment we analysed the samples by the same cDNA microarrays used before, to more widely assess the gene expression profile of the cells at this time point. By setting the threshold of regulation to two-fold, no changes in mRNA levels passing the FDR *p*-value test (*p* < 0.05) were observed in either these cDNA microarrays considered individually (the two latter cDNA microarrays were registered in ArrayExpress with accession number E-MTAB-9763 (HC11) or E-MTAB-9764 (NMuMG)). These results indicate that mammary gland epithelial cell transformation by AlCl_3_ is not initiated by direct AlCl_3_-induced regulation of specific mRNAs, and that the up- or downregulation of *Sulf2*, *Enpp5* and *Hsd11b1* observed in HC11 or NMuMG cells transformed in vitro by AlCl_3_ vs. the respective controls is a secondary event in AlCl_3_-induced cellular transformation.

### 2.3. Analysis of Genomic Instability in AlCl_3_-Transformed HC11 or NMuMG Cells by Multicolour Fluorescence In Situ Hybridization (MFISH)

In a concentration range of 10–300 μM, AlCl_3_ increases DSB in mammary epithelial cells as an early response, as assessed by counting γ-H2AX nuclear foci, a well-established marker of DSB [7]. Other groups reported similar results in embryonic zebrafish cells [11] and in *Arabidopsis thaliana* [12]. The fate of these DSBs, as well as their potential relevance to aluminium-induced cellular transformation, is not known. If not correctly repaired, they might contribute to cellular transformation by inducing genomic instability or through the formation of chromosomal rearrangements conferring a proliferative advantage. To investigate this possibility, we analysed—blind—the HC11 parental cell line and its AlCl_3_-treated derivatives (H_2_O, AlCl_3_ 10 μM and AlCl_3_ 100 μM) by MFISH, which has a resolution limit of approximately 50Mb. Any rearrangement present in more than one given condition (e.g., detected in both H_2_O and AlCl_3_ 10 μM), was considered to be pre-existing. Acentric fragments (which are unable to segregate correctly, and so are unlikely to be pre-existing in the cell line) were scored as new events. At time point zero (i.e., the passage the long-term AlCl_3_ incubation resulting in cellular transformation was started from) the HC11 parental cell line exhibits 16 identifiable chromosomal rearrangements, mainly translocations (Excel File S2A). Of these, three (i.e., translocations 3/16, 8/4, and Robertsonian translocation 14) were found uniquely in the parental cell line, each in approximately 5% of the cells analysed. At the passages used for the syngeneic injections described above, (i.e., approximately after 70 passages in culture) control (H_2_O) HC11 cells exhibit 6 unique chromosomic structural rearrangements. Compared to them, transformed HC11 cells cultured in parallel in the presence of AlCl_3_ 10 μM or AlCl_3_ 100 μM exhibit a 2.3- or 3.0-fold increase, respectively, in the number of unique rearrangements (Table 1; Excel File S2A).

**Figure 3:**
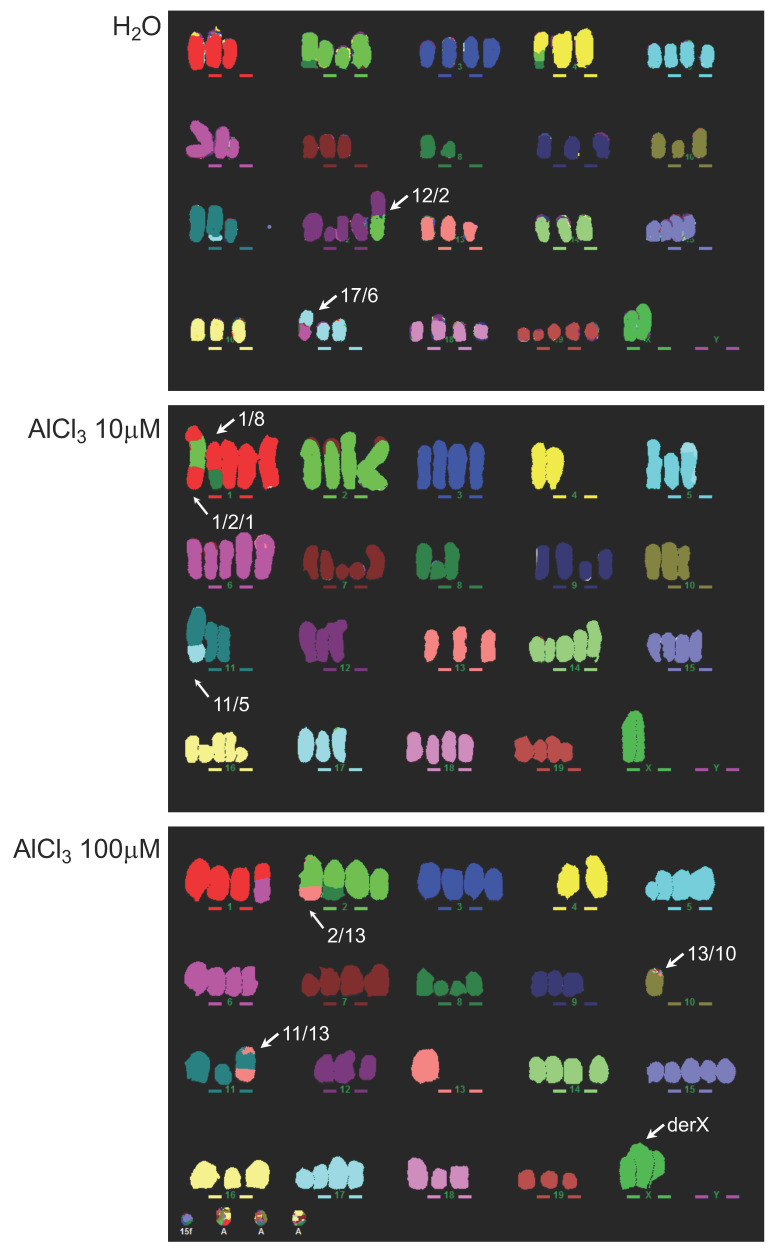
Increased chromosomal rearrangements in HC11 cells transformed in vitro by AlCl_3_. HC11 cells cultured for 71 weeks in the presence of the indicated concentrations of AlCl_3_, or the same volume (1/1000) of solvent (H_2_O) alone were analysed for the presence of chromosomal rearrangements by MFISH. The photos shown are representative examples of the results obtained. Arrows indicate chromosomal rearrangements observed uniquely in the specific condition considered. Fragments at the bottom left of the AlCl_3_ 100 μM image are pericentromeric repetitive DNA markers.

Of the rearrangements specifically present in HC11 cells transformed in vitro by AlCl_3_ 10 μM, a 1/8 translocation was present in 100% of the cells analysed (Excel File S2A). The other rearrangements observed uniquely in AlCl_3_-transformed HC11 cells occurred in a variable proportion of the cells analysed (Excel File S2A). Figure 3 shows examples of the results obtained. Analysis of variation in chromosome copy number not due to translocations revealed that no specific chromosome gain or loss was preferentially associated with long-term AlCl_3_ treatment. The highest dose of AlCl_3_ (100 μM) resulted in a population of cells lacking a clear modal number, an indication of genomic instability (Table 1, Excel File S2B).

**Figure 4:**
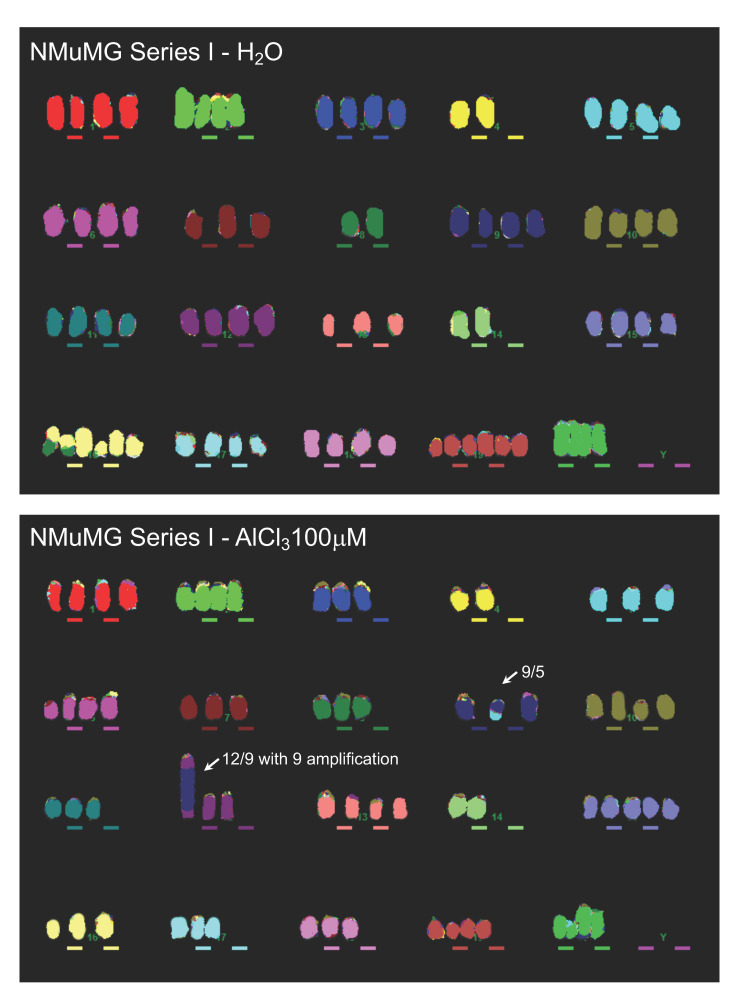
Increased chromosomal rearrangements in NMuMG cells transformed in vitro by AlCl_3_. NMuMG cells cultured for 38 weeks in the presence of AlCl_3_ 100 μM or the same volume (1/1000) of solvent (H_2_O) alone (NMuMG Series I, see text) were analysed for the presence of chromosomal rearrangements by MFISH. The photos shown are representative examples of the results obtained. Arrows indicate chromosomal rearrangements observed uniquely in the specific condition considered.

Equivalent results were obtained with NMuMG series I and III (Table 1, Excel File S2C,D). In NMuMG series I, in addition to other unique translocations, approximately 70% of NMuMG cells transformed in vitro by AlCl_3_ carry a translocation 12/9 with the amplification of chr9, as indicated by the presence of a large homogeneously staining region (HSR) on chr9 (Table 1; Excel File S2C,D; Figure 4). Figure 4 provides an example of the data obtained in NMuMG series I.

Overall, in NMuMG series I, AlCl_3_-transformed cells contain nine unique chromosomal rearrangements, compared to the five observed in untransformed controls (Table 1; Excel File S2C). In NMuMG Series III, control cultures contained 14 unique chromosomal rearrangements, whereas in cells transformed in vitro by AlCl_3_, 26 unique rearrangements were observed (Table 1; Excel File S2C,D). Of the latter, a 2/19 translocation was present in 74% of the cells analysed (Excel File S2C,D). Figure S5 provides an example of the MFISH data obtained with this series. Thus, in the three series of cells analysed, chronic culture in the presence of AlCl_3_ consistently results in an increased number of unique chromosomal rearrangements, compared to the respective controls. In addition, in three of the four AlCl_3_-transformed cells considered (HC11 cells transformed in vitro by AlCl_3_ 10 μM; NMuMG cells transformed in vitro by AlCl_3_ 100 μM of series I and series III) one such rearrangements were present in the majority of the cells analysed.

The presence of chromosome 9 amplification in AlCl_3_-transformed cells of NMuMG series I and the availability of cDNA microarray data for the same cells gave us the possibility to explore the potential functional impact of such amplification. Since MFISH is not quantitative in nature, we used quantitative real-time PCR on genomic DNA (gDNA) for a subset of genes located on chromosome 9 and up-regulated in AlCl_3_-transformed NMuMG cells of series I compared to the respective controls, to more precisely demonstrate chromosome 9 amplification in AlCl_3_-transformed cells of this series. The genes considered were *Birc2*, *Birc3*, *Cep126*, *Maml2*, *Yap1*. Of these, *Birc2* and *Birc3* are apoptosis inhibitors [15], *Maml2* is a Mastermind-like, nuclear coactivator of the *Notch* receptor occurring in salivary gland tumours as an oncogenic fusion protein [16] and *Yap1* is a well-established oncogene [17]. Their fold upregulation in AlCl_3_-transformed NMuMG cells of series I vs. controls cells in the cDNA microarray was as follows: 57.22 (*Birc2*); 34.93 (*Birc3*); 453.72 (*Cep126*); 34.16 (*Maml2*); 75.12 (*Yap1*) (FDR *p* value < 0.001 for all of them) (ArrayExpress E-MTAB-9733). By real-time quantitative PCR on gDNA, gene copy numbers for all these genes were strongly increased in AlCl_3_-transformed NMuMG cells of series I compared to untreated controls and to NMuMG parental cells (Figure S6). These genes were also found to be amplified in fresh whole cell isolates from tumours formed by AlCl_3_—transformed cells of NMuMG series I in NOD-SCID gamma mice [8] (Figure S6). Thus, in NMuMG series I, AlCl_3_-transformed cells bear gDNA amplification of several cancer genes located on chromosome 9. A concomitant, strong mRNA upregulation of the same genes was observed in the same cells. In our experiments, such amplification seems to be associated with a growth advantage in vitro and in vivo.

### 2.4. Analysis of Chromosomal Structural Abnormalities after Short AlCl_3_ Exposure

**Figure 5:**
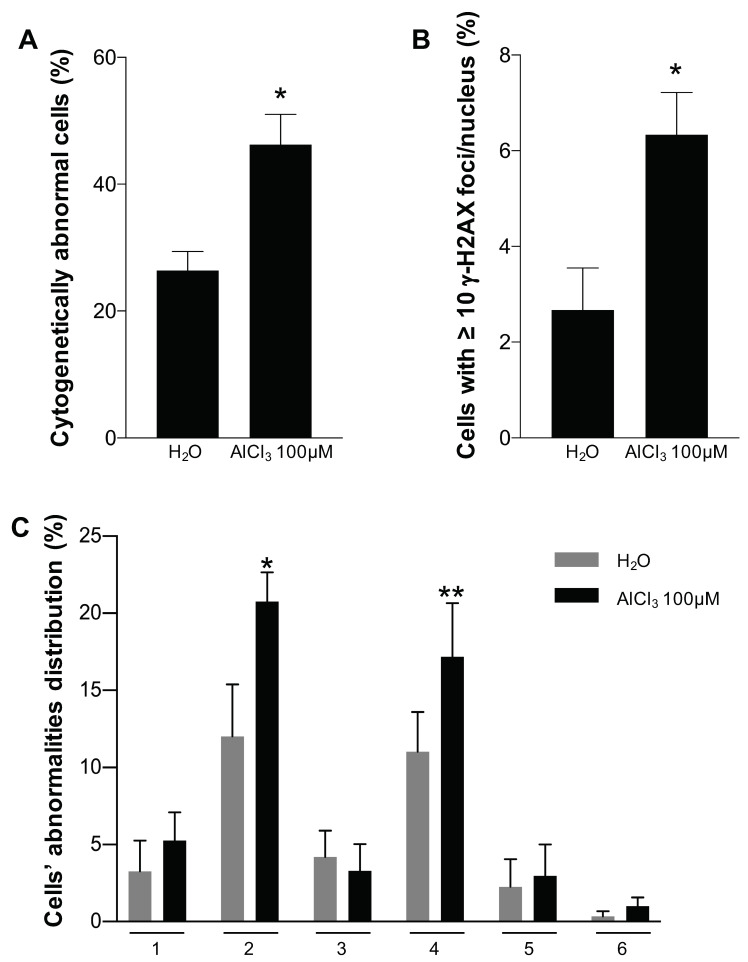
Short exposure to AlCl_3_ increases chromosomal abnormalities and γ-H2AX nuclear foci in HC11 cells. (**A**) Quantification of cytogenetically abnormal cells in HC11 parental cells incubated for 24 h in the presence of AlCl_3_ 100 μM or the same dilution (1/1000) of solvent (H_2_O) alone. The graph shows the percentage of DAPI-stained metaphases containing at least one cytogenetic abnormality. Data (mean +/− SEM) are from three independent experiments where at least 100 metaphases per condition and per experiment were evaluated blind. * *p* < 0.0001 (chi-square test); * *p*< 0.0001 (logistic regression). (**B**) Quantification of γ-H2AX immunostaining in HC11 cells incubated for 24 h in the presence of AlCl_3_ 100 μM or the same dilution (1/1000) of solvent (H_2_O) alone. The graph shows the percentage of cells with at least 10 γ-H2AX nuclear foci/nucleus. Data (mean +/− SEM) are from three independent experiments where at least 450 cells per condition and per experiment were counted. * *p*< 0.0001 (chi-square test). (**C**) Detail of chromosomal abnormalities presented in (**A**). Key: 1 = radials, 2 = DSB, 3 = premature chromosome condensation, 4 = fragmentation, 5 = telofusion, 6 = premature chromatid separation. * *p* < 0.01 (chi-square test); ** *p* < 0.05 (chi-square test).

Increased chromosome rearrangements observed in AlCl_3_-transformed HC11 or NMuMG cells vs. the respective controls by MFISH could represent a secondary effect of cellular transformation or a direct effect of aluminium. In an attempt to distinguish between these two possibilities, following a short—24 h—incubation in the presence of AlCl_3_ 100 μM, or the same dilution (1/1000) of solvent (H_2_O) alone as a control, DAPI-stained metaphases of HC11 or NMuMG parental cells were inspected blind for the presence of chromosomal abnormalities: DSB, radials, chromosome fragmentation, premature chromosome condensation (PCC), telofusion, and premature chromatid separation. We selected this concentration of AlCl_3_ because of its capacity to consistently induce mammary epithelial cell transformation in vitro ([7,8]; this paper), and because of its proximity to the aluminium concentration range measured in the mammary gland of women living in industrialized countries (0.8–87 μM) [6]. In 24-h incubations, AlCl_3_ increased the fraction of cells containing chromosomal structural abnormalities in HC11 cells, which carry a double p53 mutation [18], by approximately two-fold, compared to the controls. The difference was highly statistically significant (Figure 5A). The abnormalities induced by AlCl_3_ were mainly DSB, but chromosome fragmentations were also observed (Figure 5C and Figure S7A–C). The increase in chromosomal abnormalities in AlCl_3_-treated HC11 cells correlates with higher levels of H2AX phosphorylation in parallel cultures (Figure 5B and Figure S8A,B). In NMuMG cells, which retain p53 function [19], of 150 metaphases/conditions analysed, DSB were not detected after 24 h in AlCl_3_-treated cells nor in controls. At this time point, similar to HC11 cells, the levels of H2AX phosphorylation were higher in AlCl_3_-treated cells compared to controls (Figure S8C). DSB—as assessed by DAPI staining—were occasionally detected in AlCl_3_-treated NMuMG cultures, but not in controls, after 48 h of incubation (Figure S8D), but the numbers obtained in this cell line were too low to perform a statistical analysis.

Taken together, these experiments suggest that increased chromosomal instability observed in AlCl_3_-transformed mammary epithelial cells by MFISH results, at least in part, from a direct clastogenic effect of AlCl_3_ in this cell type.

## 3. Discussion

Aluminium constitutes approximately 8% of the Earth’s crust, where it is the third most abundant element (after oxygen and silicon) and the most abundant metal. Despite being abundant in nature, aluminium has no known biological role. Highly versatile, aluminium is used in countless industrial applications and final products, including the large majority of commercially available antiperspirants and sunscreens, where its concentration can reach approximately 1M [6,20]. In antiperspirants, aluminium prevents sweating, probably by physically blocking the pores of sweat glands through the formation of aluminium–protein precipitates. Food additives, anti-acid drugs and vaccines also contain aluminium. Thus, chronic, lifetime human exposure to aluminium is a fact in modern, industrialized society.

Like other non-essential metals [21] aluminium is acknowledged to be toxic, especially for the central nervous system, when it reaches abnormally high concentrations in the body, as it occurs in chronic renal failure [22]. Considerably less is known regarding the potential toxic effects of aluminium doses that we encounter during an ordinary life. In women living in industrialized countries, the aluminium content measured in several compartments of the mammary gland (including milk and nipple aspirate fluid) is relatively high (0.8–87 μM) compared to other compartments of the body (reviewed in [6]). The reasons for this accumulation are not known. In an overlapping range of concentrations (10–100 μM) aluminium transforms mammary epithelial cells in vitro ([7,8]; this study). The transforming effects of aluminium were preceded by an increase in DSB, a phenomenon that can be observed starting from 1 h of incubation ([7]; this study). Early induction of DSB by aluminium was also observed in primary human mammary epithelial cells [7], in embryonic zebrafish cells [11] and in *Arabidopsis thaliana* [12]. The fate of these DSBs is not known. If not correctly repaired, they might contribute to cellular transformation through the formation of chromosomal rearrangements—or other mutation types—conferring a proliferative advantage, or by producing genomic instability. Mammary epithelial cells provide a unique opportunity to follow mutations potentially resulting from aluminium-induced DNA damage and contributing to cellular transformation, since they are the only cell type currently known to be transformed in vitro by chronic aluminium exposure [7,8].

In this study, we extended our observations on the transforming effect of aluminium [7,8] to HC11 cells, a well-established model of normal mouse mammary epithelium. Cases of transformation of non-tumorigenic cells that become able to spontaneously metastasize from primary tumours formed at the site of injection in immunocompetent models are rare. For example, tumours but not metastasis were observed in BALB/c mice whose mammary fat pad was injected with HC11 cells transformed in vitro by oncogenic *NeuT*/*ErbB2* [23]. When injected subcutaneously or orthotopically, HC11 cells transformed in vitro by aluminium form aggressive tumours in the presence of an intact immune system, as in the case of real tumours. Our results with HC11 cells remarkably strengthen our previous observations in MCF-10A and NMuMG cells and highlight the carcinogenic potential of aluminium.

This study aimed at attempting to uncover the mechanisms by which aluminium initiates cellular transformation. cDNA microarrays covering the majority of the mouse transcriptome revealed consistent modulation of three genes—*Sulf2*, *Hsd11b1* and *Enpp5*—in HC11 and NMuMG cells transformed in vitro by aluminium. Of these, *Sulf-2* is an extracellular sulfatase reported as a positive regulator of oncogenic signalling pathways [24,25,26]. For *Hsd11b1* or *Enpp5*, little or no evidence of involvement in cellular transformation exists. Whereas *Sulf-2* upregulation could be hypothesized to contribute to aluminium-induced cellular transformation to some extent, NMuMG or HC11 cells exposed to aluminium for four weeks—a time period that precedes cellular transformation—by the same cDNA microarrays revealed virtually no change in mRNA expression patterns—for any gene present in the microarray. In our view, these results clearly indicate that aluminium does not initiate cellular transformation by consistently regulating specific mRNA pathways in mammary epithelial cells. Indirectly, these results also seem to rule out the possibility that AlCl_3_ initiates mammary epithelial cell transformation through epigenetic modifications, as the latter would be likely to result in changes in mRNA expression patterns.

Interestingly, by lowering the considered threshold of regulation to three-fold, in all AlCl_3_-transformed HC11 or NMuMG cells analysed by cDNA microarray, we observed consistent and significant upregulation (ranging from 3.6- to 17.3- fold) of autism susceptibility candidate (*Auts*) 2, a gene involved in neuronal development and in several psychiatric disorders (ArrayExpress accession numbers E-MTAB-9723, E-MTAB-9733, E-MTAB-9735). This observation deserves further investigation, in view of the known neurotoxic effects of aluminium [22].

Misrepaired DSBs are potential candidates for events contributing to the initiation of cellular transformation in our system, as DSB increase early after AlCl_3_ exposure ([7]; this study). We previously reported that NMuMG cells transformed in vitro by AlCl_3_ accumulate different single base substitutions compared to untreated control cells cultured in parallel [8]. However, subsequent extensive analysis of those and other whole exome sequencing data we generated did not reveal a particular profile of single base mutations in aluminium transformed cells, when considering sequence context immediately 5′ and 3′ to each mutated base (our unpublished results). Additionally, in mutagenesis tests using bacteria (Ames test), yeast (an aploid strain of *S. cerevisiae*, where one can select for mutations in the *URA3* gene) or CHO cells, all systems that mainly detect single base mutations, aluminium had little or no effect ([7] and our unpublished data). Although these results do not formally exclude that single base mutations resulting from misrepaired DSBs could contribute to aluminium-induced cellular transformation, they indicate that the potential of aluminium to induce single base changes is limited.

An alternative, common outcome for misrepaired DSB is the generation of chromosomal rearrangements. The MFISH results presented in this paper favour this possibility, since in all the mammary epithelial cell series investigated, aluminium-transformed cells consistently contained more unique chromosomal rearrangements compared to the respective controls. In most cases, the rearrangements observed were translocations, a typical outcome of misrepaired DSB. In three cases—HC11 transformed by AlCl_3_ 10 μM and NMuMG transformed by AlCl_3_ 100 μM in series I and series III—one specific rearrangement was observed in the majority of the culture—a translocation 16/8, a translocation 9/12 with chr 9 amplification, or a 2/19 translocation, respectively—showing that these rearrangements are associated with a strong proliferative advantage and perhaps contribute to it. In the case of NMuMG cells, chr9 amplification results in the overexpression of *Yap1*, *Birc2*, *Birc3*, known to contribute to cellular transformation. In HC11 cells transformed by AlCl_3_ 100 μM, there was no single major chromosomal abnormality. This might be due to the limited sensitivity of MFISH (approximately 50 Mb) or might reflect cellular transformation driven synergistically by multiple abnormalities.

Our results show, for the first time, that aluminium, similar to well known carcinogens—including arsenic or cadmium [21]—at concentrations in the range of those measured in the human mammary gland and that transform mammary epithelial cells in vitro, promotes genomic instability—in the form of chromosomal rearrangements—in mammary epithelial cells. More importantly, we show that chromosomal abnormalities likely to lead to those observed in transformed cells by MFISH—i.e., chromatide breaks—are observed after a 24 h exposure to aluminium. The latter result suggests that the accumulation of chromosomal rearrangements in AlCl_3_-transformed cells is not simply a secondary effect of cellular transformation, but the direct consequence of a clastogenic activity of aluminium in this cell type. Whereas other components might play a role in AlCl_3_-induced cellular transformation as a whole process, we propose that early induced chromosomal rearrangements might be one of the initiating effects by which aluminium transforms mammary epithelial cells.

Environmental and lifestyle factors account for 75–80% of the overall individual susceptibility to breast cancer, with the remaining fraction being ascribed to inherited factors. Among the contributing environmental/lifestyle factors are hormone exposure, alcohol consumption and obesity. However, their actual contribution to breast cancer incidence is small, leaving the majority of the cases unassigned. As a widely present element devoid of a physiological role that transforms mammary epithelial cells in vitro in the same range of concentrations measured in the human mammary gland, aluminium is a suitable candidate for the role of environmental breast carcinogen. The observation that it enables mammary epithelial cells to form highly aggressive tumours in immunocompetent animals, and that this effect is preceded by clastogenicity, a well-recognized mechanism of cellular transformation, strongly supports this conclusion.

## 4. Materials and Methods

### 4.1. Cell Culture

HC11 cells, purchased from the Leibniz-Institut DSMZ GmbH (Braunschweig, Germany) were grown as recommended by this provider. NMuMG cells, kindly provided by Prof. R. Montesano (University of Geneva) or purchased from LGC Standards/ATCC (Molsheim, France) (cat. no. CRL-1636), were grown as described [8]. For long term cultures in the presence of aluminium, every seven days, HC11 cells or NMuMG cells were seeded in 75 cm^2^ flasks at the density of 5 × 10^4^ cells/flask or 3 × 10^6^ cells/flask, respectively. AlCl_3_ was added to the medium at the time of seeding as a 1/1000 dilution of a 10 mM or 100 mM stock (resulting in a final concentration of 10 μM or 100 μM, respectively). The medium of controls received 1/1000 (*v*/*v*) of the same water used for the preparation of the AlCl_3_ stock solutions. Media and treatments were renewed after three days. AlCl_3_ stock solutions were prepared as described [7]. The cells purchased from LGC Standards/ATCC or from the Leibniz-Institut DSMZ GmbH were guaranteed to be mycoplasma-free. In addition, the mycoplasma-free status of the cells used in this study was assessed by DAPI staining.

### 4.2. Growth-In-Low-Attachment (GILA) Assay

GILA assay was performed according to published procedures [14]. Briefly, cells were seeded in 6-well low-adherent plates (cat. No. 3471, Milian, Vernier, Switzerland) in triplicate at the density of 3 × 10^4^/well and grown in 3 mL complete medium/well for 6 days. Three days after seeding, the wells received 2 additional ml of new medium/well (without removing the old medium). Six days after seeding, cell proliferation was measured by Presto Blue cell viability reagent (cat. No. A13261, Thermo Fisher Scientific (Waltham, MA, USA)). AlCl_3_ was not added to the GILA assay.

### 4.3. Syngeneic Grafts

Animal studies were conducted after being reviewed by the internal TransCure bioServices Welfare committee and approved by the French Ethical committee (CELEAG, N°101) and the French Ministry of Research and Innovation. The project was approved on the 13^th^ October 2019. The project identification code was “Tests Precliniques en oncologie sur la souris”. HC11 cells cultured for 71 weeks in the presence of AlCl_3_ 10 or 100 μM or the same dilution (1/1000) of solvent (H_2_O) alone were trypsinized and counted using an automatic cell counter. Cells were centrifuged, washed with PBS and resuspended in sterile PBS and Corning*^®^* Matrigel*^®^* Growth Factor Reduced Basement Membrane Matrix (cat. No. 354230, Corning, NY, USA) at a 1:3 ratio with 5 × 10^6^ cells/mouse for subcutaneous injection, or 1:1 ratio with 10^6^ cells/mouse for orthotopic injection as described [27]. Tumour volume and body weight were monitored weekly. Mice were sacrificed five months after injection. At dissection, tumours—or the injection site if there was no palpable tumour—were fixed in 4% formaldehyde and processed for histological analysis. The mice (6 weeks old BALB/cByJ females) were purchased from the Charles River Laboratories (Wilmington, MA, USA).

### 4.4. Ki67 Immunohistochemistry

Ki67 immunohistochemistry was performed using a rabbit polyclonal antibody (cat. No. ab15580, Abcam, Cambridge, UK) according to manufacturer’s instructions followed by K5001 LSAB+ Dako REAL Detection Systems (Agilent (Santa Clara, CA, USA) cat. No. K500111-2). Irrelevant rabbit polyclonal IgG from the same provider were used as a negative control. Ki67 proliferation index in tumours was assessed semiquantitatively by an expert breast cancer pathologist (JCT) by estimating the approximate ratio of Ki67 stained tumour cells/total number of tumour cells in all the tumours.

### 4.5. RNA Analysis

Total RNA was isolated using *mir*Vana™ RNA Isolation Kit (cat. No. AM1560, Thermo Fisher Scientific). cDNA microarrays used three independent biological replicates of each sample in the Clariom™ S Assay HT mouse system (cat. No. 902971, Thermo Fisher Scientific). Data analysis was performed by Transcriptome Analysis Console (version 4.0) (Thermo Fisher Scientific).

### 4.6. Real-Time Quantitative PCR

Reverse transcription was performed using High-Capacity cDNA Reverse Transcription Kit™ (cat. No. 4368814, Thermo Fisher Scientific). Real-time q-PCR was performed using PowerUp™ SYBR^®^ Green Master Mix (cat. No. A25777, Thermo Fisher Scientific) with the StepOne™ Real-Time PCR System (cat. No. 4376357, Thermo Fisher Scientific). Primer sequences are reported in Supplementary Materials and Methods.

### 4.7. MFISH

Chromosome spreads were prepared with standard techniques. Briefly, cells were incubated with Karyomax Colcemid (cat. No. 15210040, Thermo Fisher Scientific) 50 ng/mL for 3 h. Following a swelling step in hypotonic 75 mM KCl for 15 min, the cells were fixed twice in Carnoy’s fixative (methanol:acetic acid 3:1). The cells suspension was dropped onto clean slides, and air-dried. The MFISH was carried out with the 21Xmouse kit (cat. No. D-0425-060-DI, MetaSystems Probes, Altlussheim, Germany). A minimum of 20 images per cell type/condition were acquired and analysed with the Leica Cytovision (Wetzlar, Germany) software, on an Olympus (Tokyo, Japan) BX-51 epifluorescence microscope equipped with a JAI CVM4+ progressive-scan 24 fps black and white fluorescence CCD camera. The analyses were performed blind.

### 4.8. Quantification of γ-H2AX Foci and Chromosome Analysis after Short Aluminium Exposure

Each experiment was conducted in three independent replicates and quantitative analyses were performed blind. Cells were plated in T25 flasks for chromosome analysis, and 22 × 22 mm coverslips for γ-H2AX foci quantification, in the following way: 10^5^ HC11 parental cells/flask and 10^4^ cells/coverslip; 10^6^ NMuMG parental cells/flask and 2.5 × 10^4^ cells/coverslip. After 24 h, the medium was removed, and replaced with serum-free medium containing AlCl_3_ 100 μM or the same dilution (1/1000) of solvent (H_2_O) alone. For chromosome aberrations analysis, the cells were incubated for 24 h, as indicated before harvesting metaphase spreads, as described above. The chromosomes were mounted in DAPI/Vectashield (cat. No. H-1200, Vector Laboratories, Burlingame, CA, USA). For each cell line/condition and replicate, a minimum of 100 cells was analysed with the microscope set-up described above. γ-H2AX foci staining: 24 h after the addition of AlCl_3_ or H_2_O, the HC11 and NMuMG cells grown on coverslips were fixed in 2% formaldehyde and permeabilized in PBS containing 0.5% Triton X-100. The γ-H2AX foci were detected with mouse anti-phospho Ser139 H2AX (Millipore, clone JBW301, Burlington, MA, USA), followed by AlexaFluor 488 Goat anti-mouse secondary antibody (Thermo Fisher Scientific). The coverslips were mounted with DAPI Vectashield. Images were acquired with a Leica DM6B microscope for epifluorescence, with a DFC 9000Gt black and white fluorescence CCD camera, and operated by the Leica LASX software. Five different areas were captured with the 20× objective, with the same exposure conditions for all slides. The γ-H2AX foci were quantified using the CellProfiler software (Massachusetts Institute of Technology, Cambridge, MA, USA). The pipeline used was based on Becker et al. [28]. The diameter of nuclei was set between 20 and 70 pixels and the diameter of γ-H2AX foci between 1 and 8 pixels.

### 4.9. Data Availability

The datasets of the results presented in this study are available upon request from the corresponding author (S.J.M.).

## Figures and Tables

**Table 1:** M-FISH analysis of HC11 cells, or of NMuMG cells of Series I (S. I) or Series III (S. III) transformed in vitro by AlCl_3_ 10 μM, AlCl_3_ 100 μM or parallel control cultures incubated in the presence of the same dilution (1/1000) of solvent (H_2_O) alone, as indicated. (**a**) Weeks of treatment; (**b**) Treatment; (**c**) Modal number; (**d**) Total number of chromosomal rearrangements (CR); (**e**) Number of unique CR; (**f**) number of analysed metaphases. NA: not applicable.

Cell Line	a	b	c	d	e	f
**HC11**	0	- (parental)	77	17	3	18
	71	H_2_O	69	27	6	24
	71	AlCl_3_ 10 µM	74	34	14	25
	71	AlCl_3_ 100 µM	NA	41	18	24
**NMuMG**	0	- (parental)	39	1	1	24
	38	H_2_O S. I	67	8	5	23
	38	AlCl_3_ 100 µM S. I	72	14	9	23
	40	H_2_O S. III	64	23	14	25
	40	AlCl_3_ 100 µM S. III	65	37	26	23

## Supplementary Materials

The following are available online at https://www.mdpi.com/1422-0067/21/23/9332/s1. Reference [29] is cited in the supplementary materials.

## Abbreviations

AlCl_3_	Aluminium chloride
DAPI	4′,6-Diamidino-2-phenylindole dihydrochloride
DSB	DNA double strand breaks
γ-H2AX	phosphorylated histone H2AX
gDNA	genomic DNA
GILA	Growth-in-low-attachment
HE	hematoxylin/eosin
MFISH	Multicolor Fluorescence in situ hybridization
NMuMG	Normal murine mammary gland
PBS	Phosphate buffered saline
PCC	premature chromosome condensation
