# Supplementary material for: Genomic Instability Is an Early Event in Aluminium-Induced Tumorigenesis
Source: Int J Mol Sci. 2020 Dec 7;21(23):9332. doi: 10.3390/ijms21239332 (PMC7730342; doi:10.3390/ijms21239332)
Supplement: Supplementary file 1 [file ijms-21-09332-s001.zip › ijms-982987-for conversion-supplementary/Mandriota et al 2020 SuppMM - IJMS-R1_AN_SM.pdf]

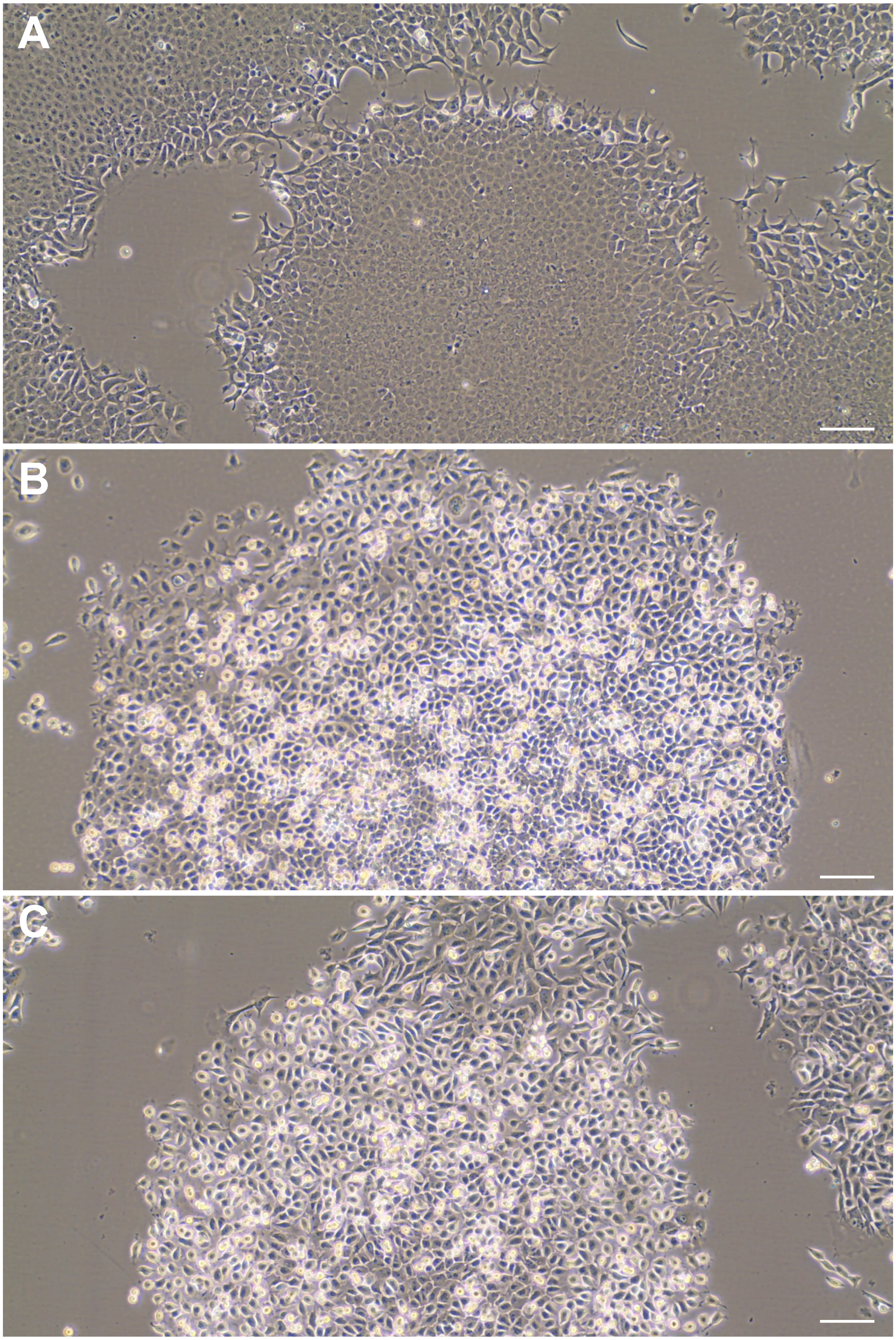

Fig. S1. Loss of contact inhibition in HC11 cells cultured in the presence of $AlCl_3$. HC11 cells cultured for 71 weeks in the presence of $AlCl_3$ 10μM (B), $AlCl_3$ 100μM (C) or the same dilution (1/1000) of solvent (H_2O) alone (A) were photographed under phase contrast. Bar = 100μm.

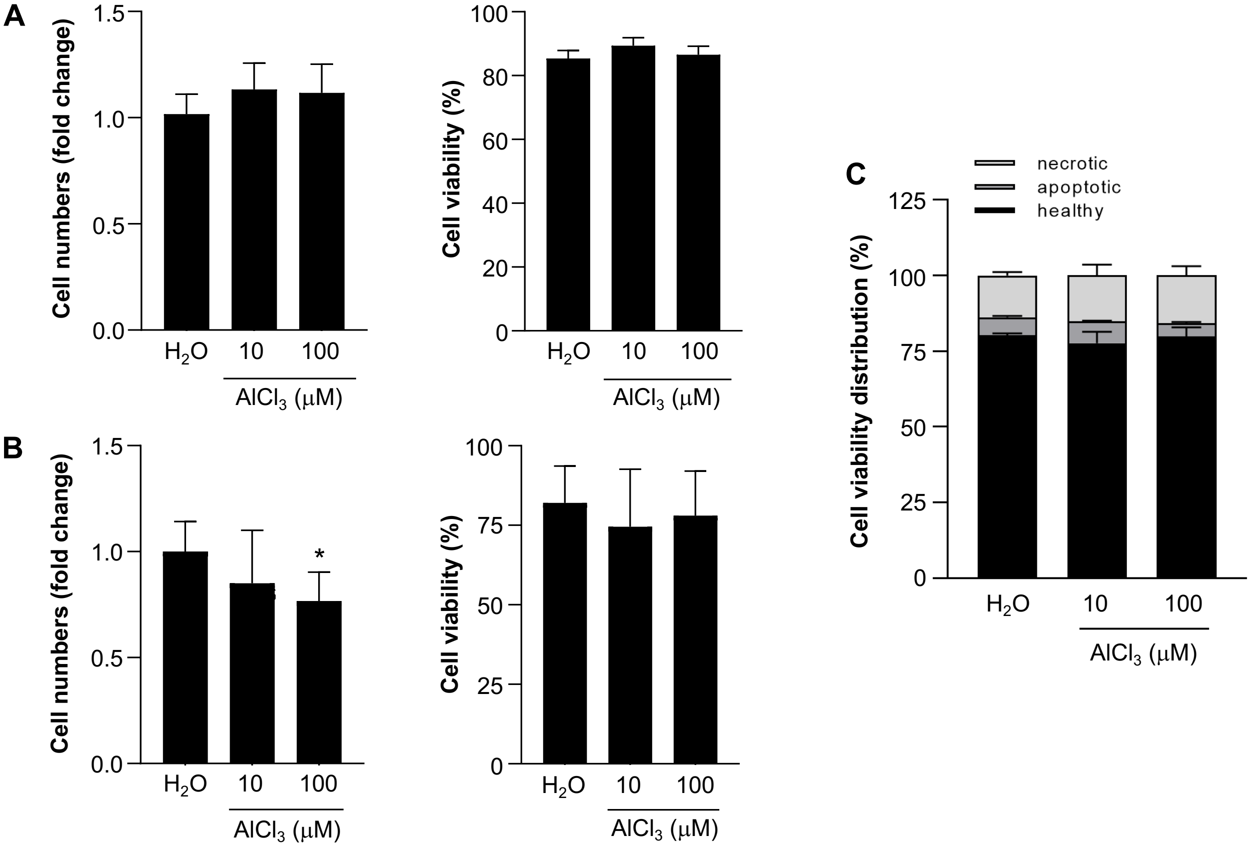

Fig. S2. $AlCl_3$ is not a mitogen for HC11 cells. **A**) Left panel: HC11 cells were seeded in 6-wells culture plates at the density of $6x10^3$ cells/well and cultured in the presence of the indicated concentrations of $AlCl_3$, or same dilution (1/1000) of solvent (H_2O) alone, for four days. At the end of the incubation, cells were counted using an automated cell counter. The values reported in the graph are means +/- SEM from three independent experiments performed in triplicate. The number of cells in controls (H_2O) was set to 1. Right panel: in the same samples, cell viability was assessed by trypan blue exclusion with an automated cell counter. **B**) Same as A, except that incubation was for seven days. Medium and treatments were renewed 4 days after seeding. Left: **p* $AlCl_3$ 100μM *vs* H_2O = 0.049 (Dunnett's multiple comparison test). **C**) HC11 cells were seeded in 6-wells culture plates at the density of $6x10^3$ cells/well and cultured in the presence of the indicated concentrations of $AlCl_3$, or same dilution (1/1000) of solvent (H_2O) alone, for four days. At the end of the incubation, cell viability was assessed by annexin V/PI staining in flow cytometry. The values reported in the graph are means +/- SEM from two independent experiments performed in triplicate.

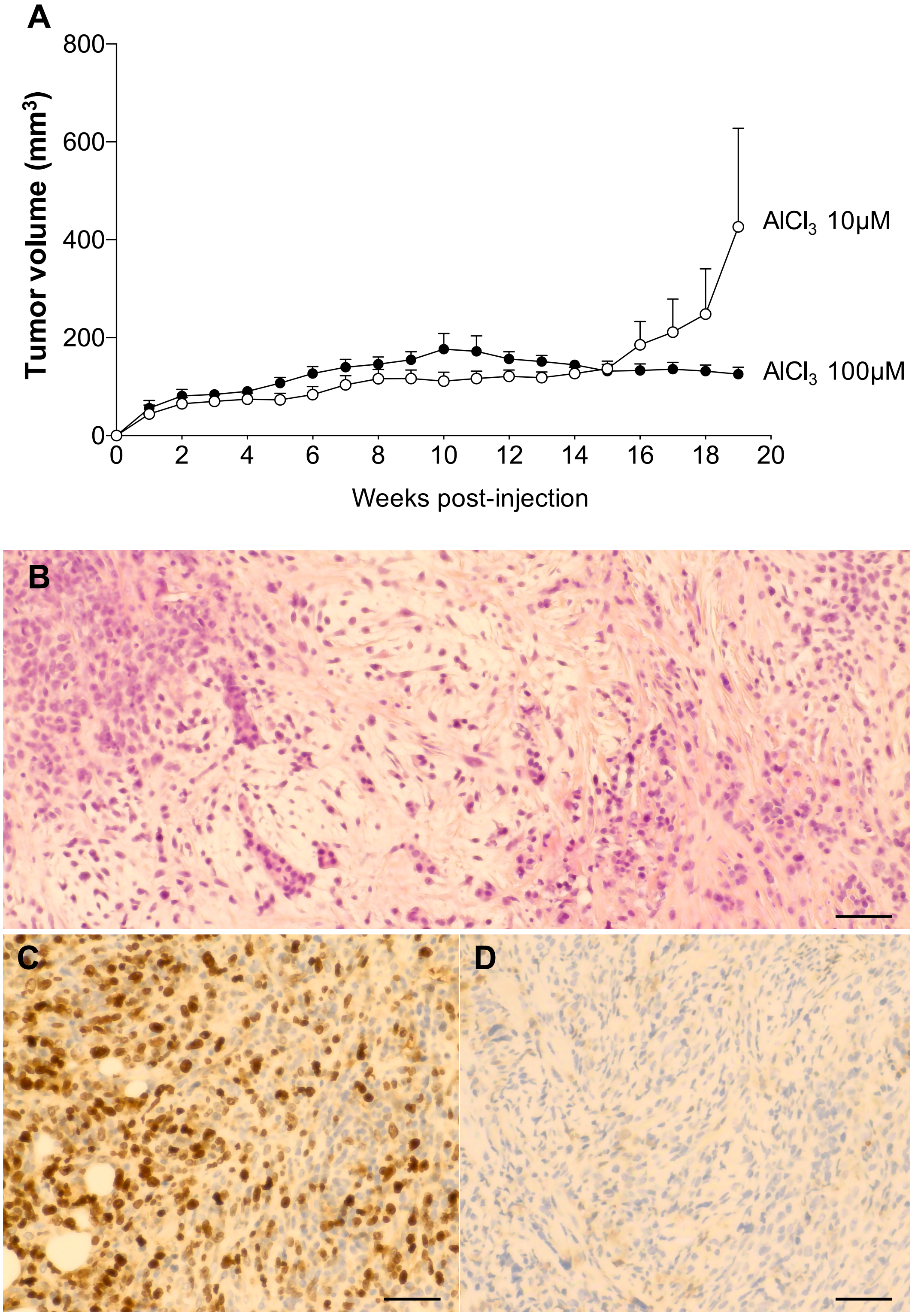

Fig. S3. Kinetics and histology of tumors formed by $AlCl_3$-transformed HC11 cells. A) The graph shows the growth, over time, of the tumors reported in Fig. 1B. Tumor size was calculated according to the formula: V (mm^3) = d^2 (mm^2) x D (mm)/2, where d and D are the smallest and largest tumor diameters, respectively, at the indicated number of weeks after sub-cutaneous injection. Results were obtained from 2 independent experiments. Values displayed are means +/- SEM. **B**) Hematoxylin/eosin staining or **C**) representative Ki67 staining or **D**) irrelevant polyclonal rabbit IgG staining of a tumor formed by HC11 cells transformed *in vitro* by $AlCl_3$ 10μM, after subcutaneous injection into a 6-weeks old BALB/cByJ female mouse. Bar = 50μm in B-D.

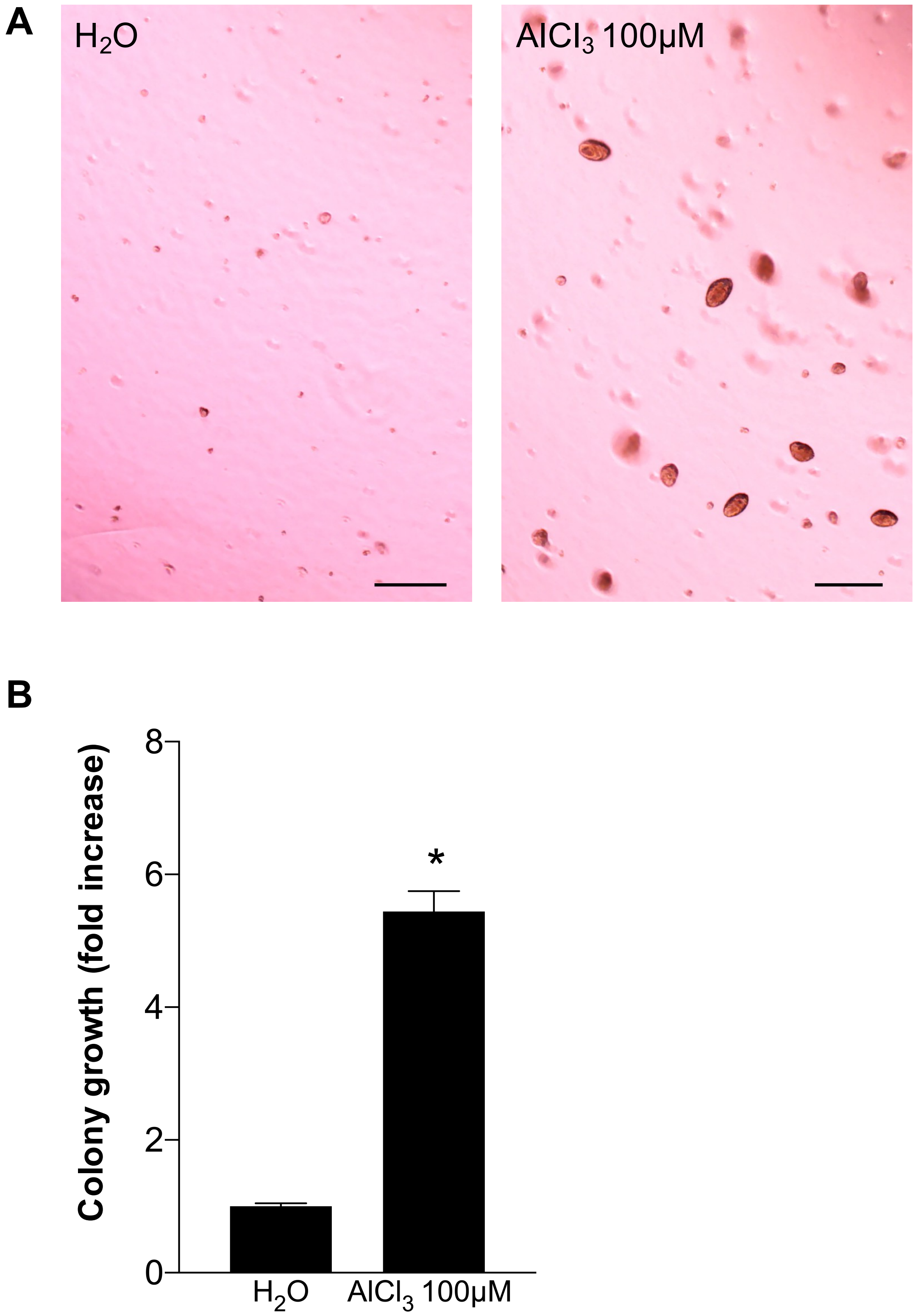

Fig. S4. A) $AlCl_3$ transforms NMuMG cells *in vitro*. NMuMG cells cultured for 40 weeks in the presence of $AlCl_3$ 100µM or the same dilution (1/1000) of solvent (H_2O) alone were grown in soft agar for 14 days in the absence of $AlCl_3$. Bar = 200µm. **B**) The growth in agarose gels was quantified by measuring the total area of colonies having an area of at least 100 pixels (corresponding to a colony of approximately ten cells). Growth of controls (H_2O) was set to 1. Colony number and area were measured in at least 17 fields in 6 different wells from 2 independent experiments per condition. The values shown are means +/- SEM. **p* $AlCl_3$ 100µM *vs* H_2O < 0,0001 (unpaired two-tailed t-test).

Excel File S1 is provided separately.

Table S1. A) ***Sulf2*****,** ***Enpp5*** **or** ***Hsd11b1*** **mRNA levels in $AlCl_3$-transformed HC11 or NMuMG cells**. mRNA levels were assessed by cDNA microarray in HC11 cells or NMuMG cells of Series I (S. I) or Series III (S. III) transformed *in vitro* by $AlCl_3$ 10μM or 100μM or parallel control cultures incubated in the presence of the same dilution (1/1000) of solvent (H_2O) alone, as indicated. FDR p-values refer to Anova.

Condition	Cell line	***Sulf2***		***Enpp5***		***Hsd11b1***	
		Fold change	*FDR p-value*	*Fold change*	*FDR p-value*	*Fold change*	*FDR p-value*
$AlCl_3$ 100μM *vs* H_2O	NMuMG S. I	+ 5.84	<0.000001	- 7.44	<0.000001	- 4.06	0.0055
	NMuMG S. III	+ 106.34	<0.000001	- 11.39	<0.000001	- 6.72	0.0005
	HC11	+ 24.86	<0.000001	- 26.48	<0.000001	- 65.14	<0.000001
$AlCl_3$ 10μM *vs* H_2O	HC11	+ 21.90	<0.000001	- 35.74	<0.000001	- 45.68	0.00003

B) Same as A, except that mRNA levels were assessed by Real-Time quantitative PCR. *nd*: undetectable. p-values refer to Dunnett's multiple comparisons test.

Condition	Cell line	***Sulf2***		***Enpp5***		***Hsd11b1***	
		Relative mRNA level ± SEM	*p-value*	*Relative mRNA level ± SEM*	*p-value*	*Relative mRNA level ± SEM*	*p-value*
$AlCl_3$ 100μM *vs* H_2O	NMuMG S. I	6.85 ± 0.38	0.0001	0.25 ± 0.02	0.000006	0.29 ± 0.11	0.0031
	NMuMG S. III	552.1 ± 7.39	<0.000001	0.15 ± 0.02	0.000005	0.24 ± 0.01	<0.000001
	HC11	20.10 ± 3.69	0.0067	0.05 ± 0.02	<0.000001	0.12 ± 0.05	0.00006
$AlCl_3$ 10μM *vs* H_2O	HC11	13.05 ± 3.45	0.0249	*nd*	-	0.10 ± 0.06	0.00009

Excel File S2 is provided separately.

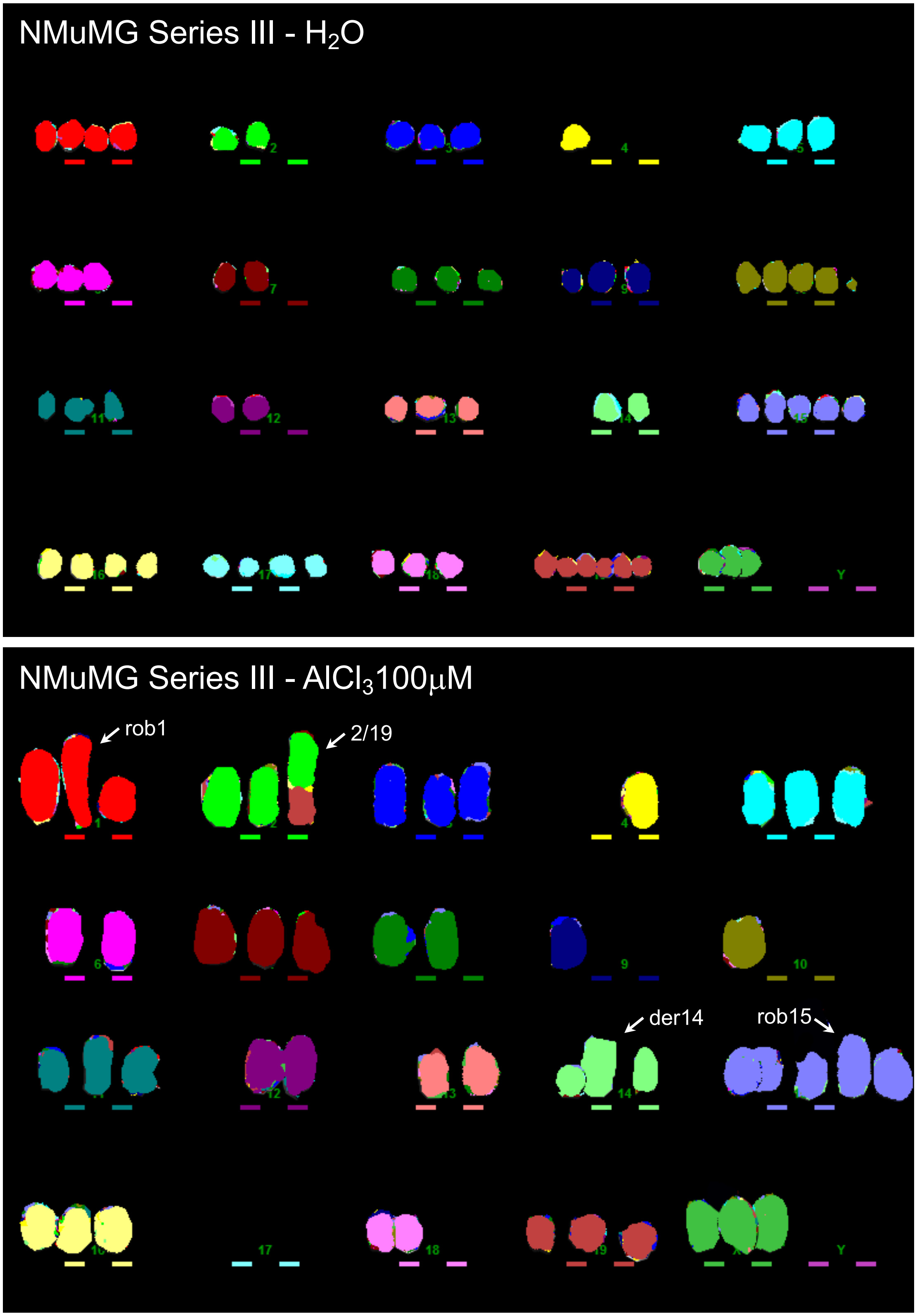

Fig. S5. MFISH analysis of NMuMG Series III. NMuMG cells cultured for 40 weeks in the presence of $AlCl_3$ 100µM or the same volume (1/1000) of solvent (H_2O) alone (NMuMG Series III, see text) were analyzed for the presence of chromosomal rearrangements by MFISH. The photos shown are representative examples of the results obtained. Arrows indicate chromosomal rearrangements observed uniquely in the specific condition considered.

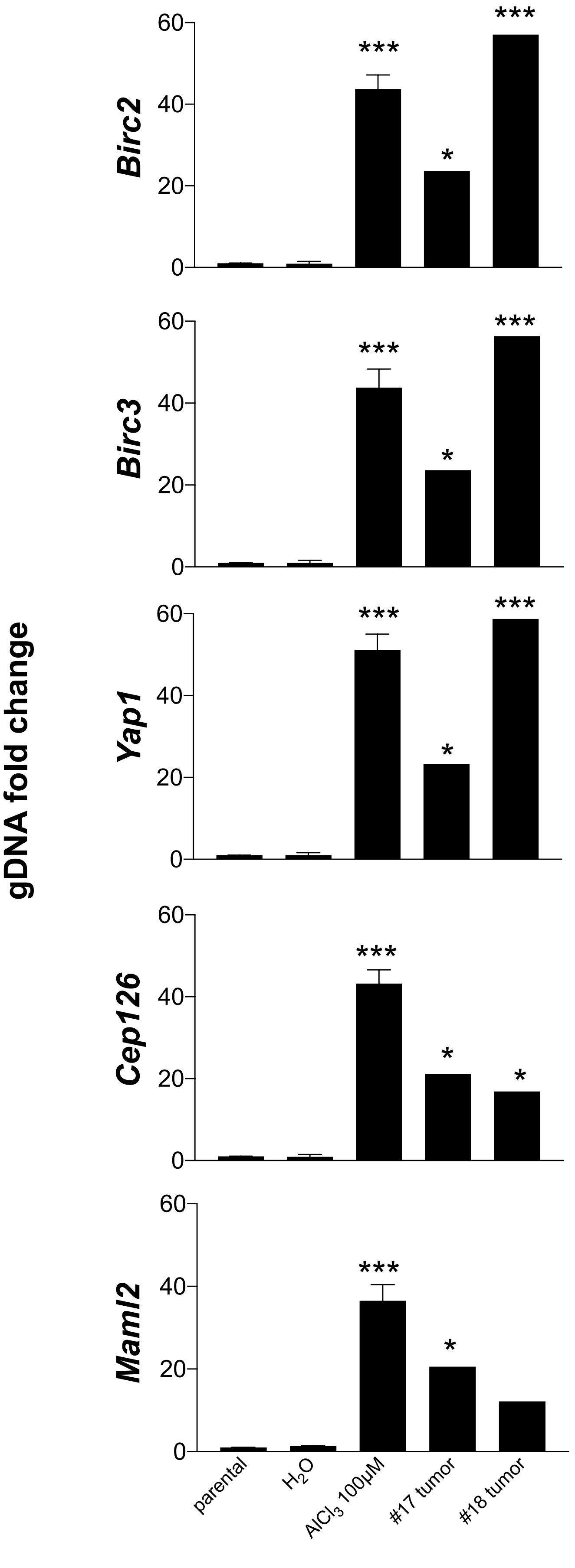
gDNA fold change
Birc2
Birc3
Yap1
Cep126
Maml2
0
20
40
60
***
*
parental
H_2O
$AlCl_3$ 100μM
#17 tumor
#18 tumor

Fig. S6. Chromosome 9 amplification in NMuMG cells transformed *in vitro* by $AlCl_3$. Genomic DNA purified from parental NMuMG cells (parental), from NMuMG cells cultured for 38 weeks in the presence of $AlCl_3$ 100μM ($AlCl_3$ 100μM) or the same dilution of solvent (H_2O) alone (NMuMG Series I, see text), or from fresh whole cell isolates from two different tumors (#17, #18) formed by $AlCl_3$-transformed cells of the same series in NOD-SCID gamma mice [8] were analysed for *Yap1*, *Birc2*, *Birc3*, *Cep126* or *Maml2* gDNA quantities by Real-Time quantitative PCR. The graphs show the average values +/- SEM from three independent biological replicates (parental, H_2O, $AlCl_3$ 100μM) or from the two individual tumors. ***$p<0.001$; *$p<0.05$ (Dunnett's multiple comparisons test).

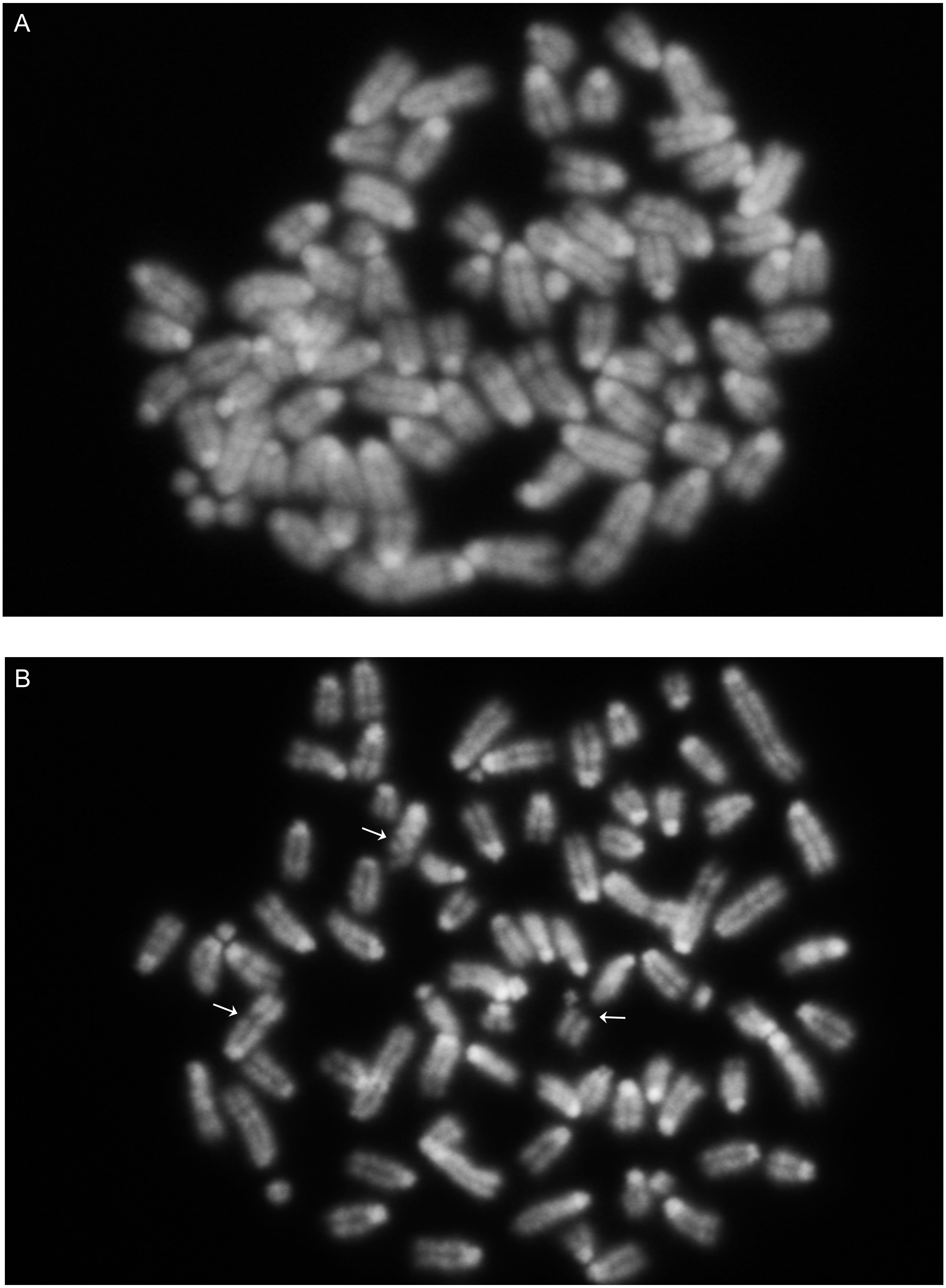
A
B

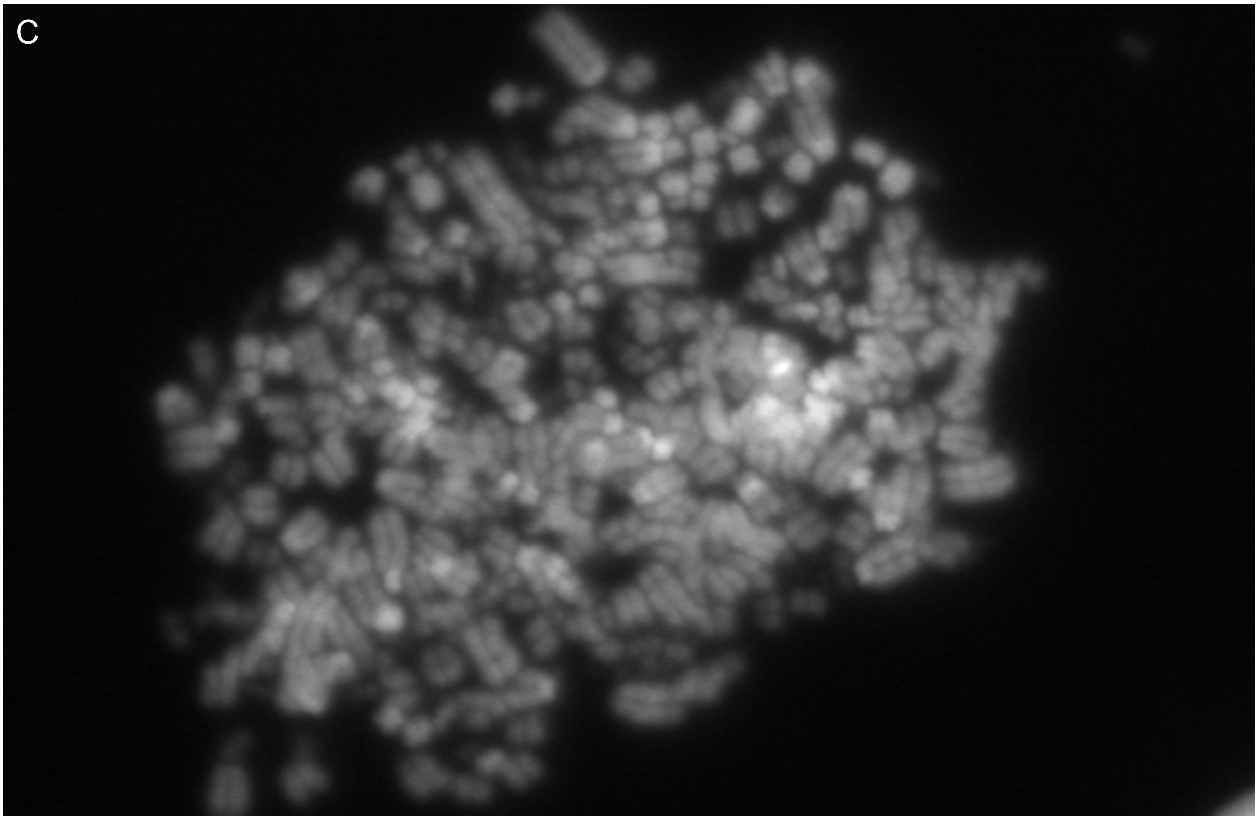

Fig. S7. Short $AlCl_3$ exposure induces chromosomal structural abnormalities in HC11 cells. DAPI-stained metaphases of HC11 cells incubated for 24 hours in the presence of $AlCl_3$ 100µM (**B, C**) or the same dilution (1/1000) of solvent (H_2O) alone (**A**). Arrows in B indicate DSB. C shows chromosome fragmentation.

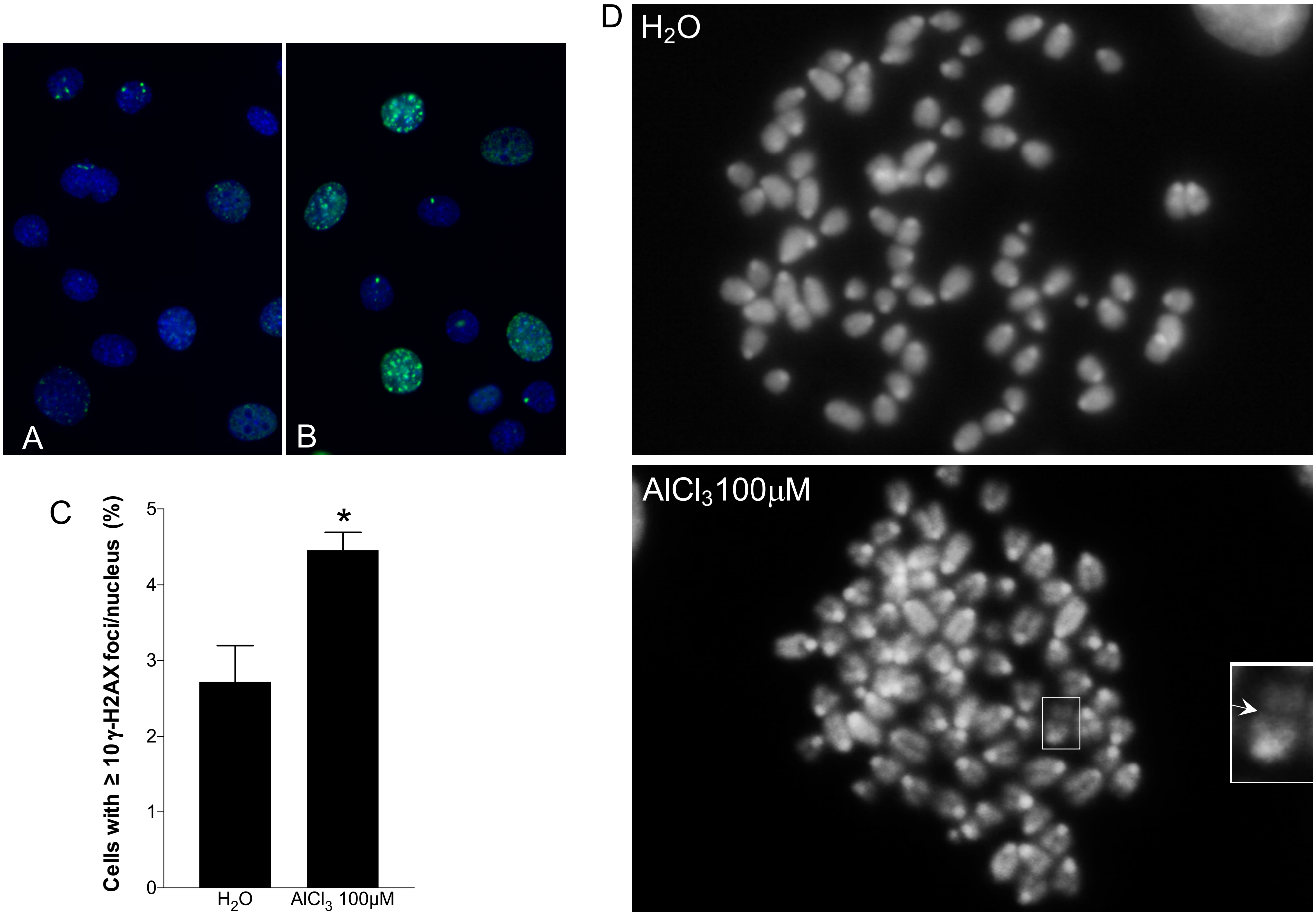

Fig. S8. Short $AlCl_3$ exposure induces DNA damage in HC11 and NMuMG cells. A, B) Examples of γ-H2AX immunostaining in HC11 parental cells incubated for 24 hours in the presence $AlCl_3$ 100μM (B) or the same dilution (1/1000) of solvent (H_2O) alone (A), whose quantification is reported in Fig. 5B. Nuclei were counterstained with DAPI. **C**) Quantification of γ-H2AX immunostaining in NMuMG cells incubated for 24 hours in the presence $AlCl_3$ 100μM or the same dilution (1/1000) of solvent (H_2O) alone. The graph shows the percentage of cells with at least 10 γ-H2AX nuclear foci/nucleus. Data (mean +/- SEM) are from three independent experiments. p=$AlCl_3$ *vs* H_2O < 0.0001 (chi-square test). **D**) DAPI-stained metaphases of NMuMG cells incubated for 48 hours in the presence of $AlCl_3$ 100μM or the same dilution (1/1000) of solvent (H_2O) alone. Arrows indicate DSB (inset).

Supplementary Materials and Methods.

Cell proliferation assay

Cells were seeded in 6-well plates in triplicate at the density of $6x10^3$ cells/well. The indicated concentrations of $AlCl_3$ or the same dilution (1/1000) of solvent (H_2O) alone were added at the time of seeding. Medium and treatments were renewed after 3 days for the 7-day proliferation experiments. Four or seven days after seeding, cells were counted with an automated cell counter that simultaneously measures trypan blue exclusion as a measure of cell viability.

Flow cytometry

Cell viability was measured by flow cytometry using an Annexin V/Propidium Iodide apoptosis detection kit (cat. No. 88-8005-72, Thermo Fisher Scientific). Stained cells were analyzed on an Attune Nxt flow cytometer (Thermo Fisher Scientific). Data analysis was performed using FlowJo software (version 10.6.1) (BD Life Sciences, Franklin Lakes, NJ, USA).

Soft agar assay

Soft agar assay was as described [7, 8]. After 14 days, colonies were photographed using an EVOS XL Core Cell Imaging System (Thermo Fisher Scientific) at 4x magnification. Quantification of the cell growth was performed by ImageJ software (version 8) (National Institute of Health, Bethesda, MD, USA) as described [29]. $AlCl_3$ was not added to the soft agar assay.

Genomic DNA extraction

Genomic DNA was extracted using PureLink™ Genomic DNA Mini Kit (cat. No. K182000, Thermo Fisher Scientific).

Real-Time quantitative PCR

Primer sequences were as follows (5' to 3'):
For mouse cDNA:
Hprt _F: GCCGAGGATTTGGAAAAAGTG; Hprt_R: TGTAATCCAGCAGGTCAGCA;
Gapdh _F: AGGTCGGTGTGAACGGATTTG; Gapdh_R: TGTAGACCATGTAGTTGAGGTCA;
Igfbp4 _F: GCAAGATGAAGATCGTGGGGA; Igfbp4_R: GTTGCGGTCACAGTTTGGAA;
Enpp5_F: GTAGCTGCCATCCTGCCAAA; Enpp5_R: GGCCACAGCTACGATTGGT;
Hsd11b1_F: TCCAGAAGGTAGTGTCTCGCT; Hsd11b1 _R : CGAGGTCTGAGTGATGTGGTT;
Sulf2 _F : GTGGAGGGGACTACAAACTGG; Sulf2_R : AGTGCGGCTTGCTAAGGTTG.

For mouse gDNA:
Hprt_F: GCTCGAGATGTCATGAAGGAGAT; Hprt_R: AAAGAACTTATAGCCCCCCTTGA;
Gapdh _F: TCCATGACAACTTTGGCATTG; Gapdh _R: CAGTCTTCTGGGTGGCAGTGA;
Birc2 _F: CTATTACGTGGATCGCAATGATGA; Birc2 _R: CCACCATCACAACAAAAGCACTT;
Birc3 _F: TGAGAATACTGGCTATTTCAGTGGC; Birc3 _R: GGGTCTGAGGGAAAGCTCG;
Yap1 _F: AGCATGTTCGAGCTCACTCCTC; Yap1 _R: AAACGGCACCCAGCTGC;
Cep126 _F: TTTTCAAAATCCAGTTCCTCCATT; Cep126_R: TCTTGAATTTGTTTTAGGGCCTCT;
Maml2_F: CTGGCACCTACAACGTCACTTC; Maml2 _R: TTTCTCTGTTGTGTCAGCTGGTTC.
